# Supplementary material for: A scientometric overview of CORD-19
Source: PLoS One. 2021 Jan 7;16(1):e0244839. doi: 10.1371/journal.pone.0244839 (PMC7790270; doi:10.1371/journal.pone.0244839)
Supplement: S1 Appendix — (PDF) [file pone.0244839.s001.pdf]

S1 Appendix

A.1 Topic top words

Top 20 words per topic, using the LDA model. Words consisting of one or two characters are filtered out. Compare with Figure A.4 for the topic intensity over time.

- **Topic 0, Public health**: “respiratory”, “infection”, “virus”, “patient”, “child”, “influenza”, “acute”, “clinical”, “viral”, “pneumonia”, “symptom”, “test”, “diagnosis”, “case”, “positive”, “detect”, “tract”, “severe”, “cause”, “study”.
- **Topic 1, Coronavirus outbreaks**: “covid-19”, “COVID-19”, “pandemic”, “health”, “sars-cov-2”, “coronavirus”, “2020”, “public”, “care”, “2019”, “patient”, “covid-19_pandemic”, “hospital”, “medical”, “emergency”, “lockdown”, “healthcare”, “public_health”, “response”, “Health”.
- **Topic 2, Clinical medicine**: “group”, “study”, “compare”, “rate”, “significantly”, “high”, “result”, “patient”, “conclusion”, “year”, “difference”, “analysis”, “control”, “score”, “significant”, “respectively”, “method”, “associate”, “mean”, “total”.
- **Topic 3, Molecular biology**: “virus”, “sequence”, “coronavirus”, “strain”, “gene”, “assay”, “sample”, “antibody”, “isolate”, “detect”, “analysis”, “study”, “protein”, “human”, “genome”, “calf”, “result”, “acid”, “detection”, “infectious”.
- **Topic 4, Public health**: “study”, “review”, “include”, “health”, “result”, “datum”, “report”, “intervention”, “search”, “evidence”, “method”, “practice”, “quality”, “research”, “risk”, “systematic”, “literature”, “conduct”, “identify”, “article”.
- **Topic 5, Coronavirus outbreaks**: “disease”, “case”, “infection”, “outbreak”, “SARS”, “transmission”, “country”, “epidemic”, “control”, “syndrome”, “respiratory_syndrome”, “severe”, “report”, “severe_acute”, “risk”, “respiratory”, “infectious”, “death”, “China”, “spread”.
- **Topic 6, Clinical medicine**: “patient”, “treatment”, “case”, “aneurysm”, “clinical”, “treat”, “lesion”, “artery”, “chest”, “stroke”, “image”, “acute”, “outcome”, “result”, “follow-up”, “imaging”, “occlusion”, “report”, “endovascular”, “complication”.
- **Topic 7, Clinical medicine**: “patient”, “surgery”, “laparoscopic”, “surgical”, “complication”, “procedure”, “undergo”, “postoperative”, “perform”, “technique”, “case”, “result”, “time”, “pain”, “group”, “method”, “repair”, “resection”, “patient_undergo”, “mean”.
- **Topic 8, Immunology**: “cell”, “infection”, “response”, “expression”, “mouse”, “disease”, “lung”, “immune”, “increase”, “role”, “effect”, “gene”, “receptor”, “study”, “tissue”, “mechanism”, “induce”, “level”, “cytokine”, “function”.
- **Topic 9, Epidemics**: “model”, “datum”, “number”, “analysis”, “time”, “result”, “network”, “different”, “base”, “method”, “dynamic”, “study”, “propose”, “social”, “epidemic”, “paper”, “approach”, “individual”, “population”, “estimate”.
- **Topic 10, Epidemics**: “method”, “result”, “study”, “device”, “pressure”, “test”, “image”, “temperature”, “flow”, “evaluate”, “forecast”, “compare”, “measure”, “high”, “tissue”, “increase”, “system”, “concentration”, “performance”, “time”.

- **Topic 11, Molecular biology**: "protein", "virus", "viral", "cell", "replication", "coronavirus", "activity", "antiviral", "membrane", "sars-cov", "domain", "infection", "structure", "host", "binding", "inhibitor", "fusion", "interaction", "hepatitis", "site".
- **Topic 12, Immunology**: "cell", "vaccine", "antibody", "mouse", "response", "infection", "immune", "antigen", "induce", "virus", "culture", "human", "line", "t_cell", "vitro", "vaccination", "immunity", "in vitro", "challenge", "recombinant".
- **Topic 13, Public health**: "disease", "drug", "development", "review", "human", "research", "treatment", "potential", "approach", "system", "provide", "clinical", "recent", "include", "develop", "discuss", "current", "technology", "application", "pathogen".
- **Topic 14, Clinical medicine**: "patient", "treatment", "clinical", "severe", "blood", "disease", "therapy", "level", "cancer", "care", "intensive", "serum", "high", "failure", "plasma", "oxygen", "risk", "unit", "heart", "outcome".

A.2 Extra figures

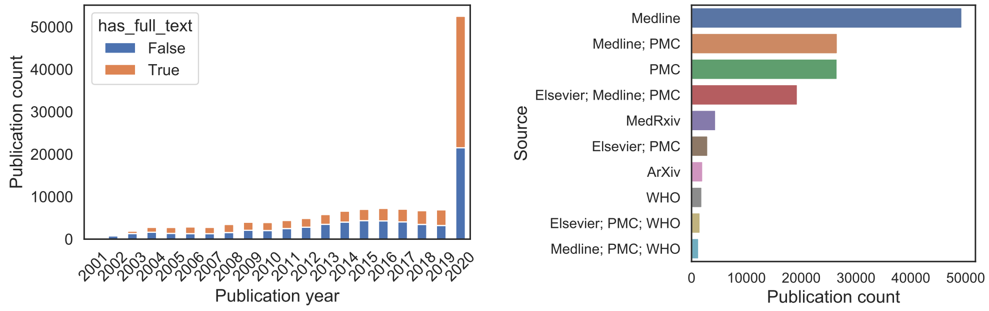

(a) Full text availability over time. **(b)** Data sources.

Fig A.1. Full text availability and data sources.

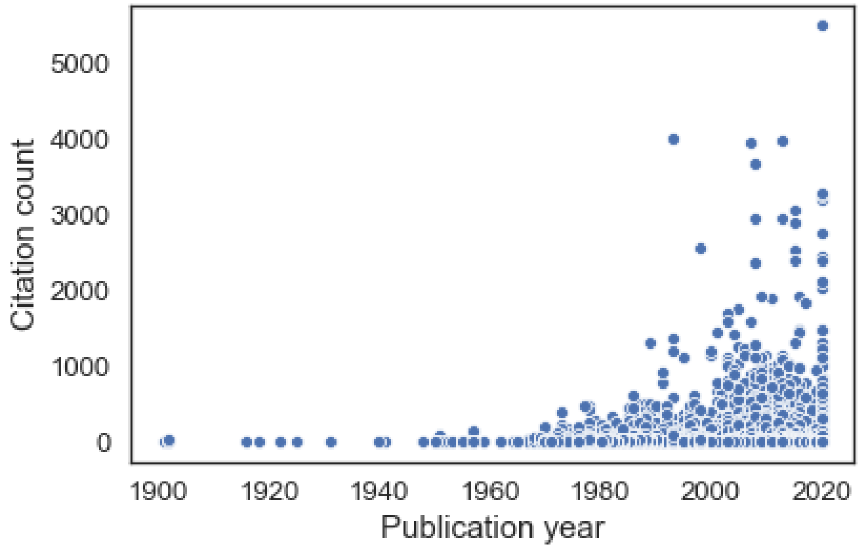

Fig A.2. Scatter plot of the number of citations received by articles from different years.

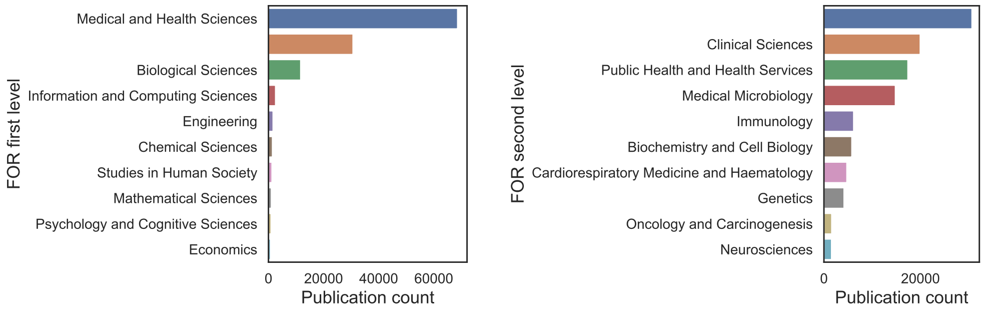

(a) First-level categories. **(b)** Second-level categories.

Fig A.3. Categories in the FOR classification in Dimensions. The empty label accounts for articles without a FOR category.

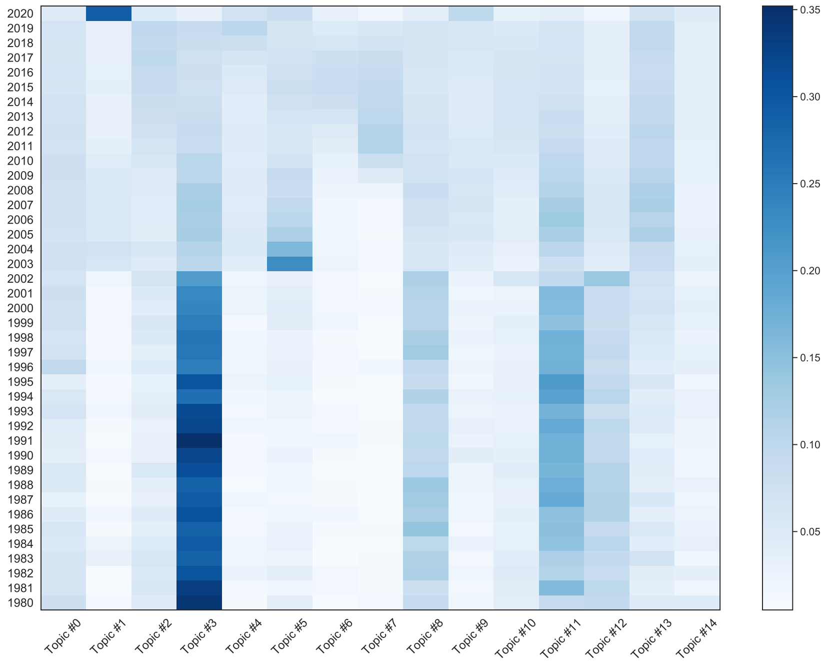

Fig A.4. Topic intensity over time, using the LDA model.

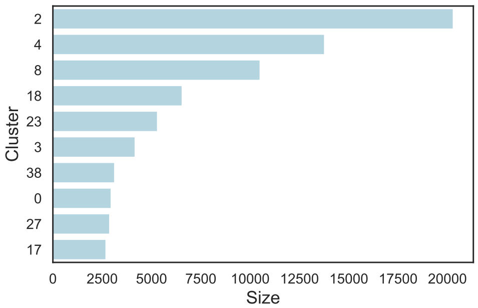

(a) Top level clustering: fewer, larger clusters.

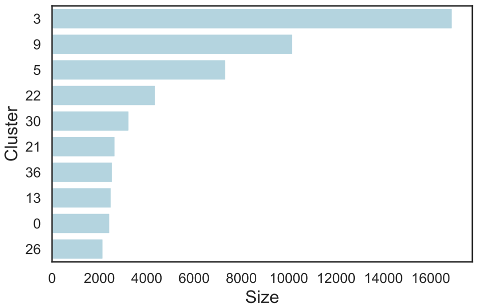

(b) Bottom level clustering: more, smaller clusters.

Fig A.5. Cluster size in the top level and the bottom level clustering of the citation network.

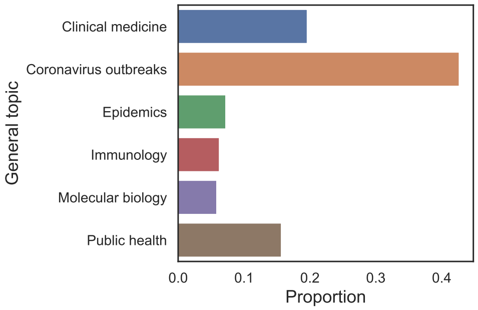

(a) Largest cluster.

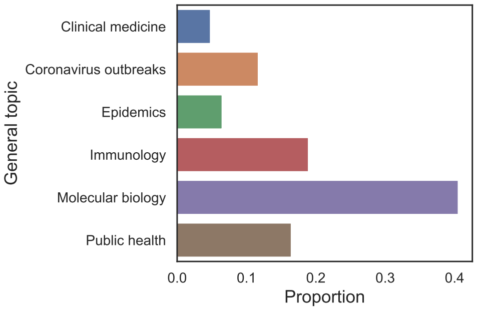

(b) Second largest cluster.

Fig A.6. Breakdown by topic for the top level clustering of the citation network.

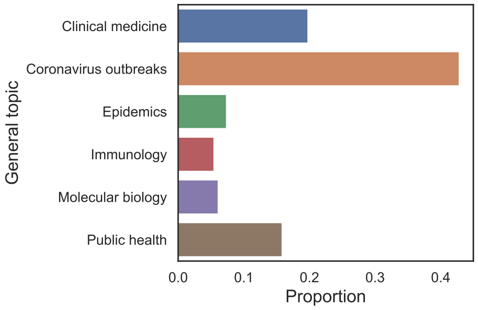

(a) Largest cluster.

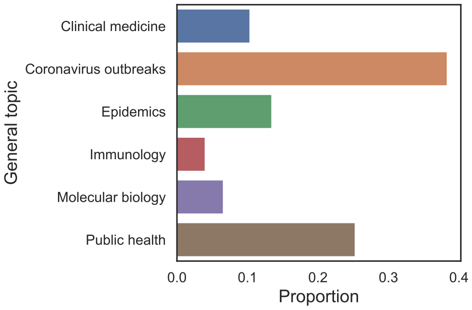

(b) Second largest cluster.

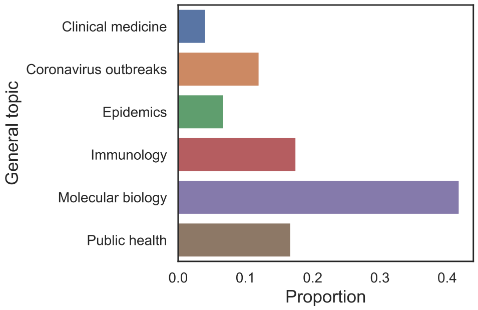

(c) Third largest cluster.

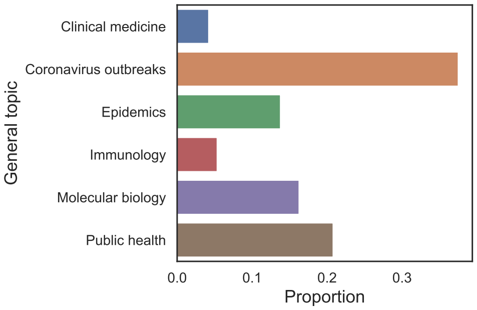

(d) Fourth largest cluster.

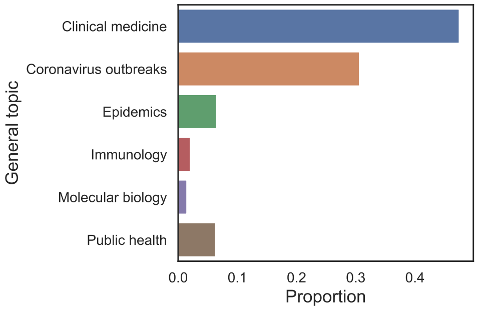

(e) Fifth largest cluster.

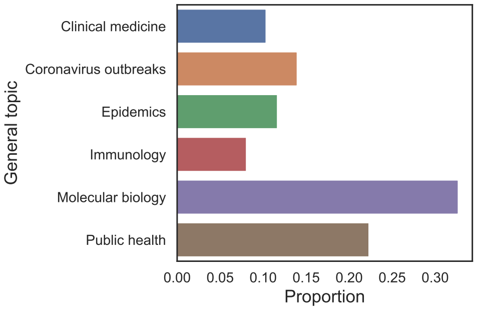

(f) Sixth largest cluster.

Fig A.7. Breakdown by topic for the bottom level clustering of the citation network.
